# Breast Cancer Polygenic Risk Score Associated with Outcomes after *In Situ* Breast Disease

**DOI:** 10.1158/1055-9965.EPI-25-0529

**Published:** 2025-10-01

**Authors:** Jasmine Timbres, Kelly Kohut, Nasim Mavaddat, Douglas F. Easton, Christos Petridis, Rebecca Roylance, Marjanka K. Schmidt, Elinor J. Sawyer

**Affiliations:** 1 Breast Cancer Genetics, King’s College London, London, United Kingdom.; 2 St George’s University Hospitals NHS Foundation Trust, London, United Kingdom.; 3 Department of Public Health and Primary Care, Centre for Cancer Genetic Epidemiology, University of Cambridge, Cambridge, United Kingdom.; 4 Department of Oncology, University College London Hospitals NHS Foundation Trust, London, United Kingdom.; 5 Division of Molecular Pathology, Netherlands Cancer Institute, Amsterdam, the Netherlands.; 6 Department of Clinical Genetics, Leiden University Medical Centre, Leiden, the Netherlands.

## Abstract

**Background:**

Ductal carcinoma *in situ* (DCIS) and lobular carcinoma *in situ* (LCIS) are preinvasive breast lesions. DCIS is treated more aggressively, as it is more likely to develop into invasive disease than LCIS. Both DCIS and LCIS face an elevated risk of contralateral breast cancer. It is thus important to identify those at high risk of further breast disease in order to personalize treatment.

**Methods:**

This study evaluated whether the 313-SNP breast cancer polygenic risk score (PRS_313_) can predict the likelihood of developing ipsilateral or contralateral breast cancer after diagnosis of DCIS or LCIS by analyzing data from patients diagnosed with DCIS (*N* = 2,169) or LCIS (*N* = 185) from the *Investigate the genetiCs of In situ Carcinoma of the ductaL subtypE* (ICICLE) and *A study to investigate the Genetics of LobulAr Carcinoma In situ in EuRope* (GLACIER) studies, with a median follow-up of 11 years. Outcomes included any further *in situ* or invasive breast disease (including distant metastasis), ipsilateral breast disease, invasive ipsilateral breast disease, and contralateral breast disease.

**Results:**

Cox regression analysis revealed a significant association between increasing continuous PRS_313_ and the risk of contralateral disease following DCIS (HR = 1.30; 95% confidence interval, 1.08–1.56) and a link between PRS_313_ and ipsilateral disease after LCIS (HR = 2.16; 95% confidence interval, 1.22–3.81).

**Conclusions:**

This research provides strong evidence that PRS_313_ can serve as a valuable predictor of future breast cancer events in women with *in situ* breast cancer, specifically contralateral disease after DCIS and ipsilateral disease after LCIS.

**Impact:**

PRS_313_ has the potential to guide clinical decisions about surveillance, risk-reduction treatments, and personalized care in those with *in situ* breast cancers, which could improve outcomes and optimize the use of healthcare resources.

## Introduction

*In situ* breast disease comprises two main distinct histopathologic entities: ductal carcinoma *in situ* (DCIS) and lobular carcinoma *in situ* (LCIS).

DCIS is the most prevalent form of preinvasive breast cancer, accounting for about 20% of all screen-detected breast cancers (1, 2). DCIS is considered a non-obligate precursor to invasive ductal carcinoma. Although not all DCIS cases will evolve into invasive breast cancer (IBC), studies have shown that, if untreated, 20% to 40% of DCIS lesions may progress to IBC over time and this is influenced by DCIS characteristics, particularly grade (3, 4). Consequently, most patients are treated with surgery, with or without radiotherapy, to prevent progression to invasive cancer, even though this approach may not always offer clinical benefit. After surgery and radiotherapy for DCIS, ipsilateral recurrence rates are approximately 13% at 10 years, with 50% to 60% of recurrences being invasive and the remainder being *in situ* (5, 6). Interestingly, recent findings suggest that 20% of ipsilateral invasive recurrences after DCIS represent new primaries, rather than true recurrences of the original lesion (7). Additionally, women with DCIS face an elevated risk of contralateral disease, with a cumulative incidence of up to 9% at 15 years (8).

LCIS is less common than DCIS, comprising ∼3% of needle biopsies (9, 10). The risk of subsequent ipsilateral invasive disease in LCIS is also lower; about 10% of women with untreated LCIS develop ipsilateral IBC (11). However, LCIS confers a higher risk of contralateral invasive disease than DCIS (12), with 40% to 50% of IBC cases after LCIS occurring in the contralateral breast (13). Therefore, LCIS is typically regarded more as a risk factor for breast cancer than a direct precursor, and women with LCIS often undergo excision biopsy to confirm that there is no evidence of invasive disease followed by increased screening. There is no attempt to completely excise the LCIS with clear margins unless pleomorphic LCIS is present. Interestingly, coexisting LCIS and invasive lobular carcinoma (ILC) share similar somatic genetic alterations (14, 15), which suggests that LCIS could also act as a precursor to invasive disease. However, not all IBC following LCIS presents as ILC; prior studies have reported that 23% to 50% of IBC after LCIS is ILC (11, 13, 16, 17).

Some risk factors that predispose to IBC also predispose to DCIS and LCIS, including family history of breast cancer, age, nulliparity, and hormone replacement therapy (18–26). It is therefore not surprising that many of the high-risk inherited breast cancer susceptibility genes (e.g., *BRCA1/2*, *CHEK2*, *PALB2*, and *TP53*) also contribute to preinvasive breast cancer (27–30).

Additionally, some of the inherited low-risk susceptibility loci identified through genome-wide association studies for IBC have been shown to be associated with DCIS and LCIS. These loci can be aggregated into a polygenic risk score (PRS), which predicts a woman’s risk of developing IBC. One of the most commonly used breast cancer PRSs is the 313-SNP breast PRS (PRS_313_) which has been shown to predict the risk of breast cancer (AUC = 0.65), particularly estrogen receptor (ER)–positive cancers (31). Notably, ∼70% of DCIS lesions are ER+ (32), and LCIS is almost always ER+ (33, 34). PRS_313_ is also associated with an increased risk of contralateral breast cancer (35), although it has not been shown to correlate with overall survival or breast cancer–specific survival after the development of IBC (36).

Although the risk factors for DCIS and LCIS are similar to those of IBC, it remains unclear whether these risk factors influence the risk of developing IBC following a diagnosis of DCIS or LCIS. Building on the previous work looking at associations with PRS_313_, this study used data from UK’s *A study to Investigate the genetiCs of In situ Carcinoma of the ductaL subtypE* (ICICLE) and *A study to investigate the Genetics of LobulAr Carcinoma In situ in EuRope* (GLACIER) case–control studies to determine associations between the PRS_313_ and outcomes in patients diagnosed with DCIS and LCIS.

## Materials and Methods

Data for this study come from three main sources: the original studies (ICICLE and GLACIER), National Health Service (NHS) Digital through the Data Access Request Service, and the Breast Cancer Association Consortium (BCAC) for PRS_313._

### Participants and data

The ICICLE study was a case–control study recruiting DCIS cases and healthy controls without a family history of breast cancer between 2007 and 2012 (ethically approved by the Southampton and South West Hampshire Research Ethics Committee A under MREC reference: 08/H0502/4). To be eligible in the ICICLE study, participants had to be diagnosed with DCIS but could have synchronous IBC in the contralateral breast. The GLACIER study (ethically approved by the Southampton and South West Hampshire Research Ethics Committee A under MREC reference: 06/Q1702/64) was a similar case–control study, focusing on the lobular breast cancer subtypes, including ILC, LCIS, and LCIS concurrent with invasive disease. Controls for GLACIER and ICICLE were often friends and nonblood relatives identified by cases and had to have no history of breast disease and no family history of breast cancer. Women were eligible for inclusion if they were diagnosed at 60 years or younger, but there were no age restrictions on controls. Cases and controls were recruited from more than 100 hospitals throughout the United Kingdom, and 3,136 DCIS cases were recruited to ICICLE, whereas 340 pure LCIS cases were recruited to GLACIER. Studies have previously been published on both the ICICLE (27) and GLACIER (37) cohorts. All participants provided written informed consent to take part in the studies, including an optional section to consent to future related research. Study activities were undertaken in line with the principles of Good Clinical Practice.

In a follow-up study from ICICLE and GLACIER (ethically approved under REC reference: 18/SW/0052 by the South West–Central Bristol Research Ethics Committee), additional data on diagnosis, treatment, and survival were requested from national cancer registries under NHS Digital for cases consenting to further research at the time of recruitment to either study. Additionally, this follow-up study was supported by the Health Research Authority on advice from the Confidentiality Advisory Group for the outcome data from NHS England, allowing for sharing patient data with NHS Digital for linkage (Confidentiality Advisory Group reference: 24CAG0139). Patient linkage was undertaken in June 2020 using NHS numbers for those for whom these data were provided, and for whom NHS numbers were missing, name, date of birth, and post code at time of recruitment were used. Follow-up data were obtained for 2,545 (81%) DCIS cases and 236 (69%) LCIS cases), with follow-up until December 31, 2019. Some patients could not be linked to records, and some did not consent to further research. Of these 2,545 DCIS cases, 329 did not have the PRS calculated, a further 23 were deemed ineligible due to age at diagnosis above 60, and 21 with contralateral invasive disease at the time of primary DCIS were excluded, leaving 2,169 DCIS cases, 35 to 60 years at diagnosis. Of the 236 LCIS cases, 42 did not have PRS calculated, 1 was excluded because of age, and 8 were excluded because of contralateral invasive disease, leaving 185 LCIS cases.

Participants in the ICICLE and GLACIER studies were required to complete a questionnaire at study entry, which included questions on reproductive, medical, and family history and use of hormones such as the contraceptive pill or hormone replacement therapies. Menopausal status was inferred from answers to questions on menstrual history and age; women still having periods were classified as premenopausal, women who stopped having periods naturally or after their ovaries were removed were classified as postmenopausal, and those that reported that their periods had stopped after treatment, contraceptive use, or hysterectomy with ovaries still remaining were only classified as postmenopausal if they were 53 or older at study entry. This method of classifying the menopausal status was based on the methodology in the Million Women Study (38). Family history of breast cancer was considered as positive if any relatives between first- and third-degree relations had a history of breast cancer. Diagnoses of DCIS and LCIS were confirmed from histopathology reports sent from the local hospitals of the participants, and from these reports data were obtained on pathology such as tumor grade, surgery type, receptor expression, and size where available. Where more than 10% data were missing, multiple imputation using chained equations was used over 40 iterations, and imputed pathology variables included tumor size, ER status, and grade.

As part of the original studies, participants were asked to provide blood samples and tumor tissue samples. The blood samples underwent genotyping using the iCOGS custom Illumina iSelect array containing more than 200,000 SNPs, as well as specific gene sequencing. PRS_313_ was obtained for most participants from BCAC, which was developed by BCAC using 94,075 breast cancer cases and 75,017 controls (SNPs included in PRS_313_ are provided in Supplementary Table S1). In the ICICLE and GLACIER patients, 52 SNPs of the 313 SNPs were genotyped as part of the original ICICLE and GLACIER studies, and the remainder were imputed against the 1000 Genomes data (31). The subtype-specific PRSs (ER−and ER+ PRS) used the same 313 SNPs as the overall PRS but allocated different weightings to SNPs that were associated with developing each subtype.

Follow-up data requested from NHS Digital included data from the cancer registry dataset comprising survival, information on diagnoses recorded under the International Classification of Diseases- (ICD) code C50 (IBC) or D05 (breast cancer *in situ*), vital status, treatment receipt including radiotherapy and systemic therapy, and hospital episode statistics providing details on surgeries. The cancer registry data held by NHS Digital did not record diagnoses of recurrent disease at the point of request, and so this was inferred from the data using methodology published by Clements and colleagues (39), with the following definitions:

Disease-free survival (DFS) was calculated as the time between first diagnosis and new occurrence of breast disease in either breast, or distant metastasis, censored at death, end of follow-up (December 31, 2019), or 10 years of follow-up time; contralateral DFS (CDFS) was calculated as the time between first diagnosis and breast disease in the opposite breast to the primary disease, censored at death, end of follow-up, or 10 years of follow-up.

Recurrence-free survival (RFS) was calculated as the time between first diagnosis and next ipsilateral breast event (either *in situ* or invasive), censored at contralateral breast events, death, end of follow-up, or at 10 years of follow-up; invasive RFS (IRFS) was calculated as the time between first diagnosis and next invasive ipsilateral breast event, censored at all other breast events, death, end of follow-up, or 10 years of follow-up. As these cases were diagnosed with *in situ* disease and not many had metastatic disease, metastatic disease was not explored as an outcome, with these cases being captured within DFS whereas RFS and IRFS recorded only local recurrences.

### Analysis

To determine whether there was a linear association between PRS_313_ and further breast disease (*in situ* or invasive), a linear regression model was fit with continuous overall PRS_313_ and then with the addition of PRS_313_^2^, and the likelihood ratio test (*P* = 0.4681) supported a linear association. PRS_313_ was separated into quartiles using the value below which 25% of participants scored, the median, and the value below which 75% of participants scored.

Kaplan–Meier survival plots were generated to inspect the difference in DFS, CDFS, RFS, and IRFS. Cox proportional-hazards regression models were fitted to determine associations between survival and PRS (both continuous and categorical in quartiles) and how these associations are affected by inclusion of other variables into the model. These variables were selected *a priori* because of the potential of confounding (DCIS grade, ER status, radiotherapy, mastectomy, family history of breast cancer, and continuous age at first diagnosis). Effect modification of family history of breast cancer was assessed by stratification of the Cox regression models and by addition of interaction terms to the model followed by determination of significance using the likelihood ratio test. The proportional-hazards assumption was tested for all Cox models using Schoenfeld residuals, and no model violated the proportional-hazards assumption. All analyses were conducted using R (v4.2.1) and Stata/MP 18.

## Results

Characteristics of 2,169 DCIS cases and 185 LCIS cases with outcome and PRS data available are displayed in Table 1. Most cases were 50 years and older at first diagnosis and were postmenopausal, 62% of DCIS cases for whom grade was available were high grade, 78% were ER+, 65% had breast-conserving surgery, 33% had a mastectomy, and 34% received radiotherapy following breast-conserving surgery. The latter two are important as mastectomy and radiotherapy will both decrease the risk of ipsilateral recurrence but have little effect on contralateral recurrence. In contrast, LCIS cases generally did not undergo mastectomy or radiotherapy. ER status is generally not assessed in LCIS, but studies have shown that the majority are ER+ (33).

**Table 1.** Characteristics and treatment of DCIS and LCIS cases with follow-up data and PRS_313_ calculated.

Characteristic	DCIS, *N* = 2,169	LCIS, *N* = 185
Age at first diagnosis (median, range)	53 (35–60)	51 (35–60)
Age at first diagnosis (group)	​	​
<50	282 (13%)	30 (16.2%)
≥50	1,887 (87%)	155 (83.8%)
Ethnicity	​	​
Mixed	0 (0%)	1 (0.5%)
Other	2 (0.1%)	0 (0%)
Unknown	4 (0.2%)	3 (1.6%)
White	2,163 (99.7%)	181 (97.8%)
Family history of breast cancer	​	​
No	1,467 (67.6%)	110 (59.5%)
Yes	702 (32.4%)	75 (40.5%)
Tumor size (mm)	19 (0.4–195)	20 (1.2–80)
Unknown	236	154
Breast surgery	​	​
Mastectomy	705 (32.5%)	21 (11.4%)
Excision	1,400 (64.6%)	145 (78.4%)
No surgery	39 (1.8%)	12 (6.5%)
Unknown/missing	25 (1.2%)	7 (3.8%)
Had radiotherapy	​	​
No	1,438 (66.3%)	178 (96.2%)
Yes	731 (33.7%)	7 (3.8%)
Mastectomy and/or radiotherapy	​	​
Neither (no mastectomy nor radiotherapy)	753 (34.7%)	157 (84.9%)
Yes (at least 1)	1,416 (65.3%)	28 (15.1%)
DCIS grade^a^	​	​
High grade	1,244 (62.6%)	0 (0%)
Intermediate grade	536 (27.0%)	0 (0%)
Low grade	206 (10.4%)	0 (0%)
Unknown	183	185
ER status^a^	​	​
Negative	239 (22.6%)	0 (0%)
Positive	870 (78.4%)	20 (100%)
Unknown	1,060	165

a Percentages reported for those without missing data.

Ten-year event rates were similar between DCIS and LCIS despite the more intensive treatment of DCIS. LCIS had a higher incidence of contralateral disease (Table 2), but this was not significantly different from the incidence in DCIS (*P* = 0.44).

**Table 2.** Follow-up data and cumulative incidence of events over the follow-up period.

Follow-up variable	DCIS, *N* = 2,169	LCIS, *N* = 185
Time period of first diagnosis	​	​
<2000	61 (2.8%)	12 (6.5%)
2000–2005	280 (12.9%)	42 (22.7%)
2005–2010	1,141 (52.6%)	104 (56.2%)
2010–2015	687 (31.7%)	27 (14.6%)
Total follow-up time before censoring at 10-year, years (median, range)	10.4 (1.04–20)	11.7 (0.76–20)
Cumulative incidence of death (any cause)	​	​
Number of events	53 (2.4%)	2 (1.1%)
10-year risk (95% CI)	2.7% (2.0%–3.5%)	1.1% (0.3%–4.3%)
Cumulative incidence of further breast disease		
Number of events	339	32
10-year risk (95% CI)	16.3% (14.7%–17.9%)	17.5% (12.7%–23.8%)
Cumulative incidence of ipsilateral breast disease (recurrence)		
Number of events	178	13
10-year risk (95% CI)	8.5% (7.4%–9.8%)	7.4% (4.4%-2.5%)
Cumulative incidence of invasive ipsilateral breast disease (recurrence)		
Number of events	80	9
10-year risk (95% CI)	4.0% (3.2%–5.0%)	5.0% (2.6%–9.4%)
Cumulative incidence of contralateral breast disease		
Number of events	120	13
10-year risk (95% CI)	5.8% (4.9%–6.9%)	7.1% (4.2%–2.0%)

**Figure 1.**
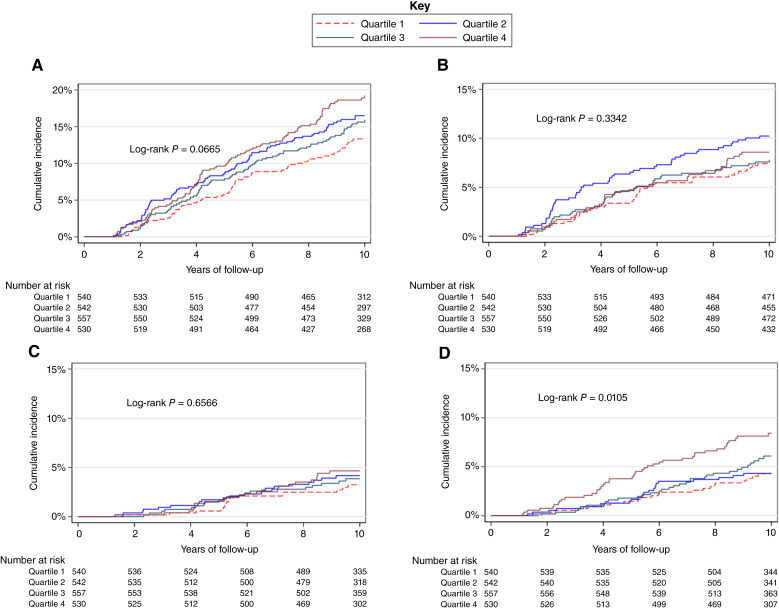
Cumulative incidence after DCIS by PRS_313_ quartiles (further breast disease, ipsilateral recurrence, invasive ipsilateral recurrence, and contralateral disease). **A,** Cumulative incidence of further breast disease after DCIS by PRS quartiles. **B,** Cumulative incidence of ipsilateral disease after DCIS by PRS quartiles. **C,** Cumulative incidence of invasive ipsilateral disease after DCIS by PRS quartiles. **D,** Cumulative incidence of contralateral disease after DCIS by PRS quartiles.

When considering PRS_313_ in quartiles, over the 10-year follow-up, there was weak evidence of an association with incidence of further breast events after DCIS and higher PRS_313_ quartiles (Fig. 1A); however, as there was no evidence of an association with ipsilateral events (Fig. 1B and C), this was likely driven by an association with increased risk of contralateral events (Fig. 1D).

Using Cox regression, continuous PRS_313_ was significantly associated with worse DFS after DCIS [HR = 1.13; 95% confidence interval (CI), 1.01–1.26], which remained unchanged after adjusting for DCIS grade, ER status, radiotherapy, mastectomy, family history of breast cancer, and continuous age at first diagnosis (HR = 1.13; 95% CI, 1.01–1.26; Table 3; Supplementary Table S2). When considering PRS_313_ in quartiles, this finding was driven mainly by an association with contralateral breast cancer relapse (HR = 1.30; 95% CI, 1.08–1.56). In regression models, the highest quartile of PRS_313_ corresponded to an increased risk of contralateral disease compared with the lowest quartile (HR = 2.03; 95% CI, 1.21–3.39) in DCIS cases after adjusting for DCIS grade, ER status, radiotherapy, mastectomy, family history of breast cancer, and continuous age at first diagnosis (Supplementary Table S3). There was no significant association with ipsilateral relapse after DCIS even when confined to invasive ipsilateral relapse (HR = 1.11; 95% CI, 0.89–1.38; Table 3; Supplementary Table S2), and the estimates were largely similar when using the ER+ PRS (Supplementary Table S4).

**Table 3.** Univariate and multivariate Cox regression including continuous overall PRS in DCIS and LCIS.

Characteristic	RFS HR (95% CI)	IRFS HR (95% CI)	DFS HR (95% CI)	CDFS HR (95% CI)
​	DCIS			
Univariate: overall PRS (continuous)	1.01 (0.87–1.17)	1.11 (0.89–1.39)	**1.13 (1.01–1.26)**	**1.31 (1.09–1.57)**
Multivariate^a^: overall PRS (continuous)	1.01 (0.87–1.17)	1.11 (0.89–1.38)	**1.13 (1.01–1.26)**	**1.30 (1.08–1.56)**
​	LCIS			
Univariate: overall PRS (continuous)	**2.15 (1.22–3.80)**	**2.50 (1.24–5.05)**	**1.43 (1.00–2.05)**	1.27 (0.75–2.15)
Multivariate^b^: overall PRS (continuous)	**2.16 (1.22–3.81)**	**2.51 (1.23–5.13)**	1.39 (0.98–1.97)	1.21 (0.73–2.02)

NOTE: Bold text indicates results with a *P* value above 0.05.

a Adjusted for ER status, DCIS grade, age at diagnosis, radiotherapy treatment, mastectomy, and family history of breast cancer.

b Adjusted for age at diagnosis and family history of breast cancer.

Multivariate analysis also showed that family history of breast cancer was associated with worse DFS after DCIS, but unlike PRS_313_ this was associated with ipsilateral recurrence (HR = 1.71; 95% CI, 1.27–2.31) and not contralateral disease (HR = 1.18; 95% CI, 0.81–1.71; Supplementary Table S2). None of the tumor characteristics expected to increase the risk of recurrence were associated with family history of breast cancer, including high-grade disease (Supplementary Table S5). As expected, mastectomy was associated with a decreased risk of ipsilateral recurrence but had no effect on contralateral recurrence (Supplementary Table S2).

**Figure 2.**
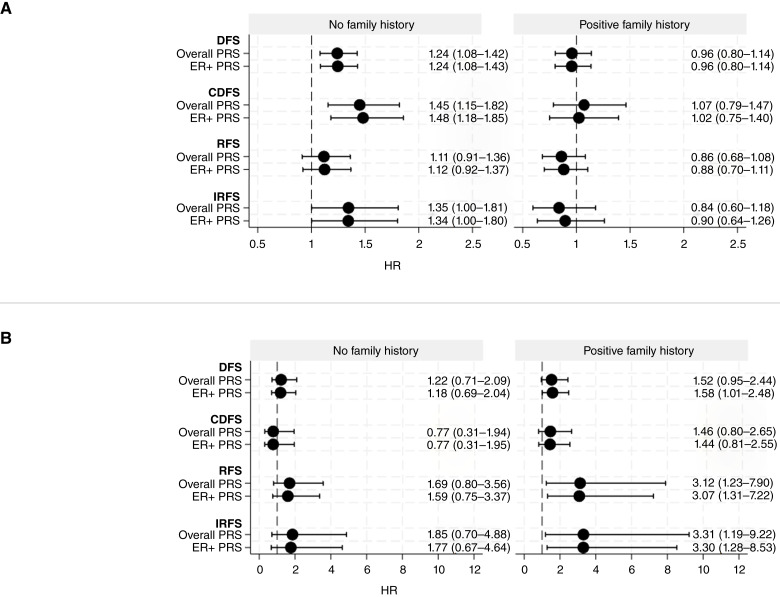
Univariate Cox regression models for PRS313 and outcomes after *in situ* breast cancer, stratified by family history of breast cancer. **A,** Outcomes after DCIS stratified by family history. **B,** Outcomes after LCIS stratified by family history.

As both PRS_313_ and family history of breast cancer were associated with worse DFS and a statistically significant interaction between PRS_313_ and family history has been shown previously (31), we stratified the Cox regression models by family history of breast cancer. When these stratified Cox regression models were examined, the stratum-specific estimates were different, with the nonstratified estimate lying between the no family history and positive family history estimates in all cases (Fig. 2A; Supplementary Table S6). This suggests that family history of breast cancer may act as an effect modifier of the association, in which case reporting stratified analysis results is preferable. Using interaction terms to investigate this modifying effect with likelihood ratio tests also showed evidence of effect modification (*P* = 0.039 in association with invasive RFS).

In those with no family history of breast cancer, statistically significant associations were found between PRS_313_ and DFS, CDFS, and IRFS after DCIS, but no significant associations were observed in those with a positive (first–third degree) family history of breast cancer. Among the women with no family history of breast cancer, after excluding those who had undergone mastectomy or radiotherapy after lumpectomy (which will reduce the risk of local recurrence), PRS_313_ was associated with an estimated 40% increase in ipsilateral disease risk (HR = 1.40; 95% CI, 1.00–1.95) with increasing PRS_313_ (Supplementary Table S7).

**Figure 3.**
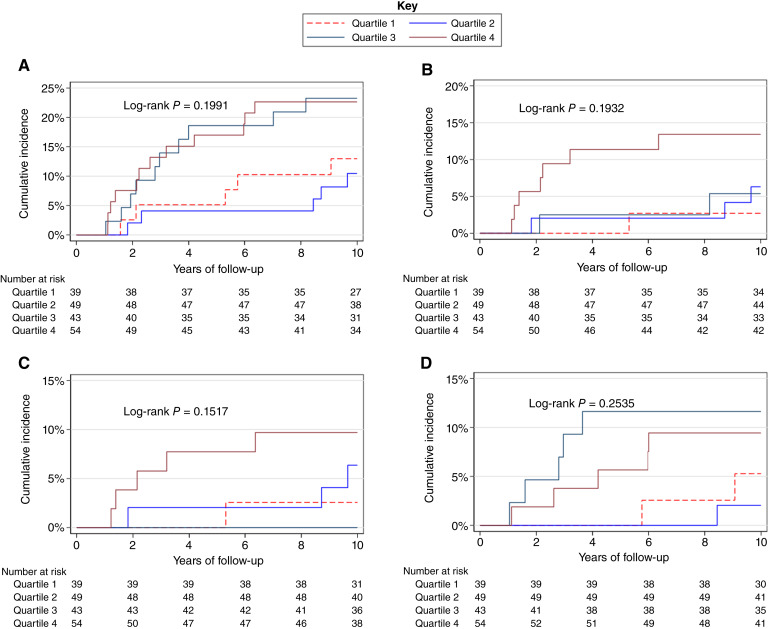
Cumulative incidence after LCIS, by PRS_313_ quartiles (further breast disease, ipsilateral recurrence, invasive ipsilateral recurrence, and contralateral disease). **A,** Cumulative incidence of further breast disease after LCIS by PRS quartiles. **B,** Cumulative incidence of ipsilateral disease after LCIS by PRS quartiles. **C**, Cumulative incidence of invasive ipsilateral disease after LCIS by PRS quartiles. **D**, Cumulative incidence of contralateral disease after LCIS by PRS quartiles.

Within the LCIS cases, those in the highest PRS_313_ quartile had a higher cumulative incidence of ipsilateral recurrence after LCIS, specifically invasive ipsilateral disease, although there was no statistically significant difference between quartiles in any of the outcomes on log-rank test (Fig. 3A–D).

Using Cox regression, continuous PRS_313_ was significantly associated with worse DFS after LCIS (HR = 1.43; 95% CI, 1.00–2.05), but this association weakened slightly after adjusting for family history of breast cancer and age at first diagnosis (HR = 1.39; 95% CI, 0.98–1.97; Table 3; Supplementary Table S8). Unlike DCIS, there was a clear association between PRS_313_ and RFS after LCIS on both univariate (HR = 2.15; 95% CI, 1.22–3.80) and multivariate Cox regression (HR = 2.16; 95% CI, 1.22–3.81), showing a greater than 2-fold increased risk of RFS after LCIS with increasing PRS_313_. In addition, this association was larger when looking at IRFS after LCIS (HR = 2.50; 95% CI, 1.24–5.05; Table 3; Supplementary Table S8). The association with CDFS after LCIS was not statistically significant, but numbers were small in this group.

On multivariate analysis, there was no evidence of an association between family history and DFS after LCIS, although the HRs were in the same direction as in DCIS. Despite this, Cox regression models were also stratified on family history of breast cancer in order to determine if there was any effect modification in LCIS, as was seen with DCIS (Fig. 2B; Supplementary Table S9).

Here, associations were only statistically significant in those with a positive family history of breast cancer. Increasing PRS_313_ was associated with a >3-fold increased risk of both any ipsilateral (HR = 3.12; 95% CI, 1.23–7.90) and ipsilateral invasive diseases specifically after LCIS (HR = 3.31; 95% CI, 1.19–9.22) but only in those with a positive family history (Fig. 2B; Supplementary Table S9). After excluding few cases that had mastectomy or radiotherapy, this association increased further to a fourfold increased risk for ipsilateral disease after LCIS with increasing PRS_313_ (HR = 4.19; 95% CI, 1.47–11.92) in those with a positive family history of breast cancer (Supplementary Table S10).

## Discussion

We have demonstrated that PRS_313_ is an independent predictor of future *in situ* or invasive breast disease after DCIS and LCIS. PRS_313_ was significantly associated with invasive contralateral disease, but not ipsilateral disease, after DCIS. This finding aligns with a previous study that also identified a statistically significant link between the PRS and contralateral disease following IBC (35). An association with ipsilateral disease after DCIS emerged when the analysis was restricted to individuals without a family history of breast cancer and excluded those who had undergone mastectomy or radiotherapy. This finding mirrors studies in IBC, which also demonstrated attenuation of the PRS in individuals with a family history of the disease (31).

As clinical trials continue to evaluate the safety of active surveillance (without surgery) for low-risk DCIS, more women could potentially opt to forego treatment. Our results suggest that PRS_313_ could be a useful tool for identifying those at higher risk of further breast disease, thus informing decisions about the suitability of active surveillance, especially given that approximately 20% of ipsilateral invasive events after DCIS are new primaries rather than true recurrences (7). Given that PRS_313_ was designed to predict the risk of IBC, it is likely that it could also help predict the risk of new ipsilateral primaries, as well as contralateral new primary disease. However, in order to distinguish true recurrences from new primaries, genomic analysis on paired primary and recurrent tumors is necessary.

The association between PRS_313_ and contralateral disease after DCIS is strong, with the highest PRS_313_ quartile having an HR of 2.03 (95% CI, 1.21–3.39). Currently, women in the United Kingdom with DCIS are recommended to undergo yearly mammograms for 5 years after diagnosis (40), after which women older than 50 years are typically transitioned to mammograms every 3 years. However, our data show that only about 40% of contralateral disease occurrences happen within the first 5 years after primary *in situ* disease, a finding also previously observed in a cohort of 107 patients with lobular neoplasia or atypical ductal hyperplasia (41). Consequently, individuals in the highest PRS_313_ quartile may benefit from extended annual screening to 10 years. Furthermore, tamoxifen or aromatase inhibitors could be considered more for those at higher risk of contralateral second primary cancers, as studies have shown that the benefits of 5 years of tamoxifen in the prevention setting persist for at least 11 years after treatment cessation (42).

In contrast to DCIS, and despite small sample sizes, we observed a clear association between PRS_313_ and ipsilateral recurrence in LCIS, which was stronger in women with a family history of breast cancer. This finding may be explained by the presence of variants in the *CHEK2* gene within PRS_313_ that correlate with the *CHEK2* c.1100delC variant, a founder mutation that has been shown to be strongly associated with LCIS and ILC (29, 43), although not universally found in all studies of ILC (44). This could also be related to a high proportion of our LCIS cases involving surgical margins after excision. We also compared the risk of invasive recurrence in DCIS with that in LCIS, as it would be expected that DCIS cases would have a higher risk, but did not find a significant difference, again possibly due to the difference in surgical management between the two groups (Supplementary Table S11). Surprisingly, in LCIS, we did not observe a statistically significant association with contralateral disease, possibly due to the small sample size. Further investigation in larger cohorts is needed.

The lack of association with ipsilateral disease after DCIS may be due to the relatively low incidence of ipsilateral recurrence, as most cases underwent surgery. This could also be attributed to only 20% of ipsilateral recurrences after DCIS being new primaries (7). Additionally, despite some studies finding characteristics such as tumor grade and size to be associated with recurrence after DCIS, we did not find such associations (Supplementary Table S12).

The key strength of this study lies in leveraging the ICICLE and GLACIER case–control studies, which were originally designed to investigate genetic predisposition to DCIS and LCIS, and unlike other cohorts of *in situ* disease with long-term outcome data, genotyping data derived from germline DNA from peripheral blood samples are available. However, the study has some limitations. The number of pure LCIS samples is small, and the analysis is based on extending the original case–control studies into a single cohort using follow-up data. Although the exclusion of some cases from follow-up due to lack of consent may introduce selection bias, this is unlikely, as the demographics of nonconsenting or untraceable participants were similar to those who participated.

In conclusion, we have shown for the first time that PRS_313_ is associated with outcomes following *in situ* breast cancer. In clinical practice, PRS_313_ could be valuable in identifying patients at higher risk for second primaries in either breast following *in situ* disease. These individuals may benefit from risk-reduction therapies such as tamoxifen or an aromatase inhibitor, as most lesions express ERs and PRS_313_ could also help determine the optimal duration for annual surveillance. Although the association between PRS_313_ and ipsilateral disease after DCIS was not statistically significant (possibly due to treatment effects in the cohort), the association observed after LCIS suggests the potential value for PRS_313_ in predicting ipsilateral disease in active surveillance studies.

## Supplementary Material

Supplemental Table 1 Table S1. Genotyped and imputed (not genotyped) SNPs

Supplemental Table 2 Table S2. Univariate and multivariate Cox regression models for recurrence-free survival (RFS) and disease-free survival (DFS) after DCIS and continuous overall PRS.

Supplemental Table 3 Table S3. Univariate and multivariate Cox regression models for overall PRS quartiles and recurrence-free survival (RFS), disease-free survival (DFS), and contralateral disease-free survival (CDFS) after DCIS.

Supplemental Table 4 Table S4. Univariate and multivariate Cox regression models for recurrence-free survival (RFS) and disease-free survival (DFS) after DCIS and continuous ER-positive PRS.

Supplemental Table 5 Table S5: Univariate logistic regression models for associations between family history of breast cancer and characteristics normally associated with recurrence in DCIS cases.

Supplemental Table 6 Table S6. Univariate Cox regression models for PRS and outcomes after DCIS, stratified by family history of breast cancer.

Supplemental Table 7 Table S7. Univariate and multivariate Cox regression models for overall PRS and outcomes after DCIS, in those with no family history of breast cancer, and excluding those treated with radiotherapy or mastectomy (N=520).

Supplemental Table 8 Table S8. Univariate and multivariate Cox regression including continuous overall PRS in LCIS.

Supplemental Table 9 Table S9. Univariate Cox regression models for PRS and outcomes after LCIS, stratified by family history of breast cancer.

Supplemental Table 10 Table S10. Univariate and multivariate Cox regression models for overall PRS and outcomes after LCIS, in those with a positive family history of breast cancer, and excluding those treated with radiotherapy or mastectomy (N=64).

Supplemental Table 11 Table S11. Univariate and multivariate Cox regression models comparing risk of developing invasive ipsilateral recurrence after DCIS or LCIS.

Supplemental Table 12 Table S12. Univariate logistic regression models for association between tumour characteristics and ipsilateral recurrence (in-situ or invasive) after DCIS.

## Data Availability

Data supporting the results of this study are held by King’s College London. Access to data from the ICICLE and GLACIER studies can be requested through BCAC: https://www.ccge.medschl.cam.ac.uk/breast-cancer-association-consortium-bcac/data-data-access. To request access to individual-level BCAC data, please follow the procedure below: (a) Download the concept analysis application form, and return the completed form to the BCAC Data Access Coordination Committee (DACC). The DACC review all submitted applications three times a year and will be in contact with the concept proposer once this review process has taken place. (Note: The BCAC data dictionary can be downloaded here). (b) If the DACC approves the concept analysis, (i) there is a one-month period for BCAC principal investigators to opt out of any analysis for their study samples, (ii) a nonnegotiable data transfer agreement (DTA) must be signed by the concept proposer and their institution, or (iii) a fee is payable for the data access. Please also refer to the concept analysis application form for more details. (c) Once the above steps have been completed, the requested data will be sent to the concept proposer, with details of the BCAC authorship/funding/acknowledgment requirements for any publications resulting from the data analyses. The current BCAC publication guidelines can be found here. If you have any queries, please contact the BCAC Coordinator. This work uses data that have been provided by patients and collected by the NHS as part of their care and support. These data are collated, maintained, and quality assured by the National Disease Registration Service, which is part of NHS England. Access to the data was facilitated by the NHS England Data Access Request Service.
